# Human Dialyzable Leukocyte Extract Enhances Albendazole Efficacy and Promotes Th1/Th2-Biased Lymphocyte and Antibody Responses in Peritoneal Cavity of Murine Model of *Mesocestoides vogae* Infection

**DOI:** 10.3390/ijms26146994

**Published:** 2025-07-21

**Authors:** Gabriela Hrčková, Dagmar Mudroňová, Katarína Reiterová, Serena Cavallero, Ilaria Bellini

**Affiliations:** 1 Institute of Parasitology of the Slovak Academy of Sciences, 04001 Košice, Slovakia; reiter@saske.sk; 2 Department of Microbiology and Immunology, University of Veterinary Medicine and Pharmacy, 04001 Košice, Slovakia; dagmar.mudronova@uvlf.sk; 3 Department of Public Health and Infectious Diseases, Sapienza, University of Rome, 00185 Rome, Italy; serena.cavallero@uniroma1.it (S.C.); ilaria.bellini@uniroma1.it (I.B.)

**Keywords:** immunotherapy, human dialyzable leukocyte extract, albendazole, lymphocytes, antibodies, parasite *Mesocestoides vogae*, mice, peritoneal cavity

## Abstract

Human leukocyte extract (HLE), a non-immunogenic dialyzable leukocyte preparation (<10 kDa), may serve as a safe adjuvant in immunotherapy. We investigated the effects of albendazole (ABZ), HLE, and their combination in *Mesocestoides vogae* infected mice, focusing on lymphoid cells in the peritoneal cavity, the site of larval proliferation and parasite-induced immunosuppression. Peritoneal lymphoid cells were analysed by flow cytometry and qPCR. Cells proliferative responses to ConA, LPS, and parasite excretory/secretory (E/S) antigens, cytokine production (ELISA), IgM and IgG isotypes in exudates and parasite antigen recognition (Western blot) were assessed. Efficacy was measured by larval burden and *14-3-3* gene expression in larvae. HLE combined with ABZ enhanced larval clearance and suppressed *14-3-3* gene expression in larvae. HLE and combination therapy increased CD3^+^ T cell frequencies, especially CD3^+high^, reduced regulatory CD3+/IL-10 Tregs and expression of *Foxp3^+^*. All treatments diminished CD19^+^/IL-10^+^ Bregs, correlating with lower *CD9* and *Atf3* mRNA levels compared to infected mice. Transcription factors *T-bet* expression was strongly upregulated, while *GATA3* was moderately elevated. IFN-γ production and T/B cell proliferation were restored after HLE and combination therapy, partially, even in the presence of E/S antigens. IgM and total IgG levels against parasite antigens declined, while Th1-associated IgG2a increased in ABZ+HLE and HLE-treated groups. Albendazole failed to reverse the immunosuppressive Treg-type immunity but was more effective in reducing Breg populations and their functions. HLE enhanced ABZ efficacy by restoring Th1 responsiveness, reducing Treg/Breg activity, and modulating antibody profiles. It represents a promising immunomodulatory adjuvant in the treatment of the infections associated with Th2/Treg-driven immunosuppression.

## 1. Introduction

The immune system plays a pivotal role in defending against infectious and parasitic diseases in both humans and animals. Traditional therapy typically relies on chemotherapeutic interventions involving chemically synthesised drugs. In recent decades, however, immunotherapy has garnered considerable attention, and research is increasingly focused on the effects of bioactive molecules derived from natural sources. Mixtures of low molecular weight molecules (up to 10 kDa), obtained from disintegrated white blood cells of humans or animals through dialysis and ultrafiltration, have demonstrated beneficial immunomodulatory properties without inducing immunogenicity. These preparations have shown therapeutic potential in the treatment of various diseases [1].

Commercially available human dialysable leukocyte extracts (hDLEs) in countries such as Mexico, Cuba, the Czech Republic and China have exhibited promise at diseases, characterised by impaired cell-mediated immunity, including viral [2,3], bacterial [4,5], and parasitic infections [6,7,8]. In the murine breast cancer model, the hDLE preparation Immodin alleviated paclitaxel-induced toxicity, reduced tumour growth, and increased lymphocyte counts, decreased myeloid-derived suppressor cells in both the blood and spleen [9]. Similarly, hDLE preparation, Transferon™ (Instituto Politecnico Nacional, Mexico City, Mexico) exhibited anti-neoplastic effects and significantly reduced brain metastases in a murine model of prostate cancer [10]. Furthermore, the administration of hDLE in a study involving 143 patients with sepsis was associated with an increased survival rate [11]. Positive outcomes have also been reported in patients with multiple sclerosis [12], chronic fatigue syndrome [13], and allergic rhinitis [14].

Infectious diseases caused by parasitic protozoa and helminths remain significant health and economic burdens for humans, livestock, and companion animals worldwide. These conditions are classified among the so-called neglected tropical diseases (NTDs), as they predominantly occur in tropical regions and disproportionately affect socioeconomically disadvantaged populations and domestic animals. Among the NTDs, an important subgroup comprises zoonotic larval cestodiases induced by flatworms [15]. The larval stages of flatworms *Echinococcus multilocularis*, *Echinococcus granulosus*, and *Taenia solium* causing cysticercosis are of particular concern. Although *Mesocestoides vogae* (syn. *Mesocestoides corti*) rarely infects humans [16], its adult stage commonly parasitises carnivores such as dogs and cats. Rodents serve as intermediate hosts for the metacestode stage, known as the tetrathyridium, which exhibits an exceptional capacity for asexual proliferation. This characteristic is also observed in the metacestodes of *Echinococcus* and *Taenia* species, reviewed by [17,18]. Rapid progression of *M. vogae* larvae in the liver and peritoneal cavity of laboratory rodents [19] makes it a valuable experimental model in pharmacological studies.

For the treatment of echinococcosis in humans, benzimidazole-based anthelmintics such as albendazole (ABZ) or mebendazole are employed, either as monotherapy or in combination with other agents [20,21,22]. However, treatment extending over several years is frequently associated with adverse effects including bone marrow suppression, gastrointestinal disturbances, proteinuria, and hepatotoxicity [23]. Beyond their antiparasitic properties, these benzimidazoles have also demonstrated anticancer activities [24]. Given that long-term treatment of these chronic infections is typically parasitostatic rather than parasiticidal [22,23], and may negatively impact the immune system, combining anthelmintic therapy with an appropriate immunotherapeutic agent represents a rational and potentially more effective approach.

A notable feature of tissue-dwelling flatworm larvae is their ability to persist in the host for extended periods despite an active immune response. This reflects their sophisticated capacity to modulate host defence mechanisms. In mice, infection with *M. vogae* larvae initially elicits a transient Th1-type response in the spleen, liver, and peritoneal cavity. Over time, this response shifts towards Th2 and T regulatory (Treg) profiles, both at the cellular and humoral levels, thereby promoting chronic infection likely due to parasite-derived excretory/secretory (E/S) products playing central role in immune modulation [25,26]. The proliferative larval stages of *M. vogae* in the peritoneal cavity of rodents constitutes a distinct inflammatory model, wherein larvae directly interact with various immune cell populations. This specific immunological milieu presents a valuable model for investigating antiparasitic efficacy and immunomodulation following the administration of pharmacological agents and biomolecules, both in vivo and ex vivo.

Our study, based on a flatworm-induced model of peritoneal inflammation, was designed to explore the effects of human dialysable leukocyte extract (HLE) and albendazole (ABZ), administered either individually or in combination. The investigation focused on the differential immunomodulatory effects of each therapy on the phenotypic and functional changes in lymphocyte T and B subsets related to Th1, Th2, and Treg-type immunity, as well as to the production of peritoneal IgM and IgG subclass antibodies against immunogenic somatic and E/S larval antigens. These immunological parameters were then correlated with the observed reduction in larval burden.

## 2. Results

### 2.1. Phenotypic Analysis of Lymphoid Sub-Populations in the Peritoneal Cavities of Mice

**Figure 1:**
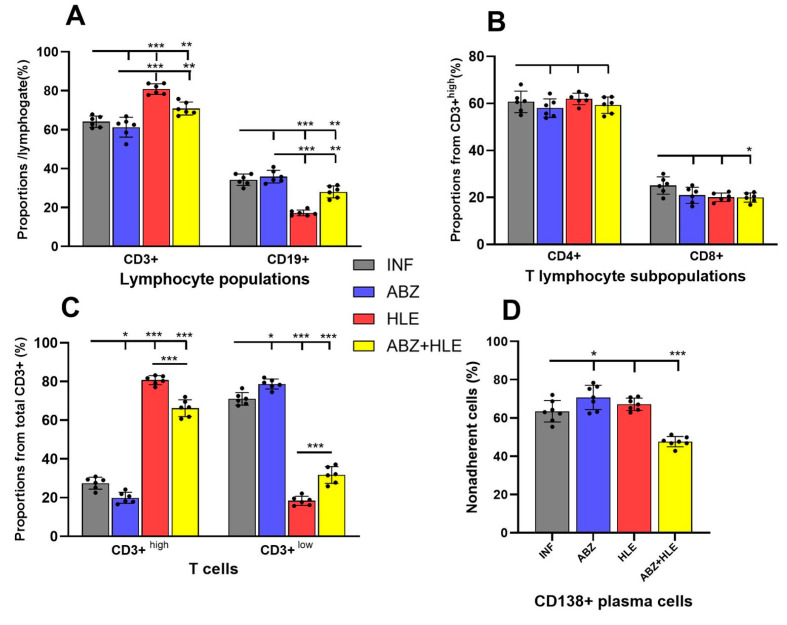

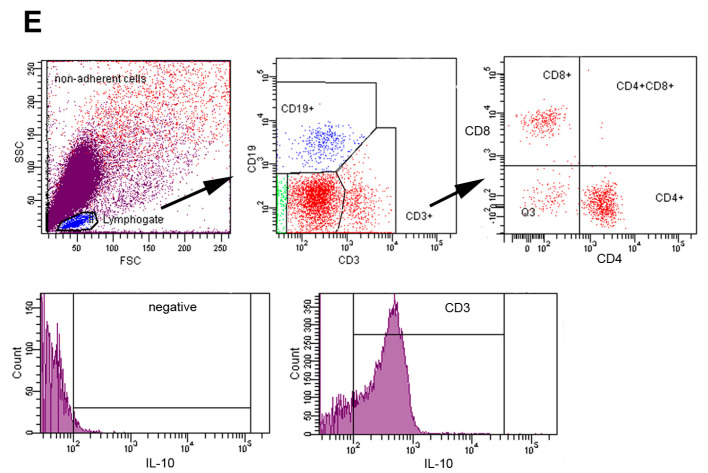
Phenotypic characterisation of lymphoid subpopulations in the peritoneal cavity of mice infected with *M. vogae* and treated with albendazole (ABZ), human leukocyte extract (HLE), or their combination. (**A**) Proportion of total CD3^+^ T lymphocytes in the peritoneal exudate. (**B**) Frequency of CD3^+high^ T cells, indicating enhanced TCR/CD3 complex expression. (**C**) Relative proportions of CD4^+^ helper and CD8^+^ cytotoxic T lymphocyte subsets. (**D**) Proportions of antibody-secreting CD19^+^/CD138^+^ plasmablasts and CD19^−^/CD138^+^ plasma cells within the non-adherent lymphoid population. (**E**) Gating strategy. Data represent mean ± SD (*n* = 7); * *p* < 0.05 ** *p* < 0.01, *** *p* < 0.001 vs. infected group.

In this study, we investigated whether ABZ, HLE, and their combination modulate the proportions and functions of T and B lymphocytes, as well as plasma cells and plasmablasts, representing up to ~40% of total peritoneal exudate cells (PECs). Non-adherent cells recovered after 18 h of cultivation contained a small proportion of granulocytes, mainly eosinophils, as confirmed by Giemsa-stained smears, reflecting their limited adherence to plastic. Flow cytometric analysis of non-adherent cells revealed that within the lymphocyte gate, the frequency of mature CD3^+^/CD19^−^/CD138^−^ T cells exceeded that of CD3^−^/CD19^+^/CD138^−^ B1 cells, the main B cell population in the murine peritoneal cavity. Compared to infected controls, HLE and combination therapies led to an increase in total CD3^+^ T cells (Figure 1A), particularly those expressing high levels of CD3 (Figure 1B). Since the CD3 complex acts as a co-receptor of the TCR [27], this suggests enhanced functional activity. Treatment did not affect the proportions of CD4^+^ helper or CD8^+^ cytotoxic T cells (Figure 1C).

Antigen-driven activation of B cells yielded large, short-lived CD19^+^/CD138^+^ plasmablasts and terminally differentiated CD19^−^/CD138^+^ plasma cells, which dominated the non-adherent fraction (Figure 1D). A significant reduction in these populations was observed only after ABZ+HLE treatment (*p* < 0.001).

### 2.2. HLE and Combination Therapy Reduce the Frequency of IL-10-Producing T and B Lymphocytes

**Figure 2:**
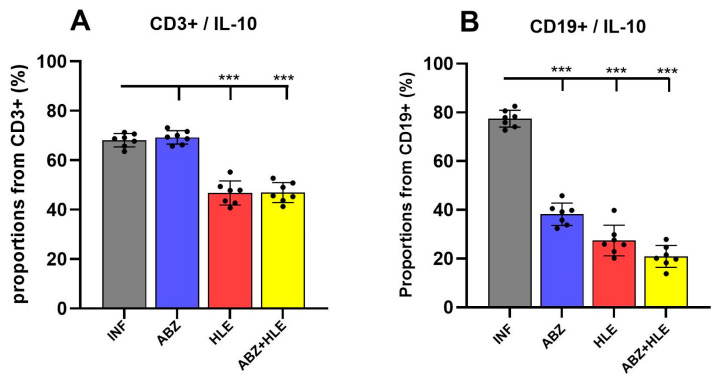
IL-10–producing regulatory T and B cells in the peritoneal cavity of *Mesocestoides vogae* infected mice following therapy. (**A**) Proportions of CD3^+^IL-10^+^ regulatory T cells and (**B**) Frequencies of IL-10–producing CD19^+^ B1 cells (Bregs). Data represent mean ± SD from flow cytometric analysis (*n* = 7); statistical comparison is indicated by connecting lines and significant difference is at *** *p* < 0.001.

Regulatory B cells (Bregs) and effector B cell subsets differentiation pathways are based on the priming signals received [28]. Breg-derived plasmablasts and plasma cells, as well as helminth-derived E/S products—particularly those rich in saccharides—can stimulate IL-10 production, promoting both Breg and Treg regulatory activities [29,30]. Flow cytometric analysis revealed that over 60% of CD3^+^ T (Figure 2A). In contrast, HLE alone or in combination with ABZ significantly cells in infected mice expressed IL-10, a pattern similarly observed following ABZ treatment (reduced the proportions of IL-10^+^ CD3^+^ T cells (*p* < 0.001), indicating attenuation of Treg-mediated suppression. Similarly, IL-10–producing CD19^+^ B1 cells were significantly diminished in all treated groups compared with infected controls (*p* < 0.001) (Figure 2B), suggesting suppression of infection-induced Breg responses.

### 2.3. Gene Expression of Transcription Factors Regulating Th1/Th2/Treg Immunity

**Figure 3:**
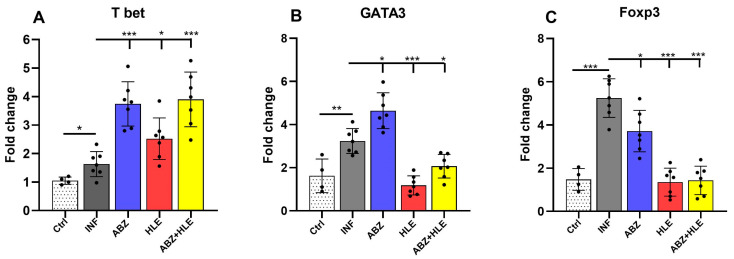
Modulation of transcription factors defining Th1, Th2 and Treg responses in peritoneal lymphoid cells. mRNA expression levels of (**A**) *T-bet*, (**B**) *GATA3*, and (**C**) *Foxp3* were analysed in peritoneal lymphocytes by qPCR following termination of therapy corresponding to day 26 p.i. Data are shown as mean ± SEM (means of triplicate analysis) (*n* = 7); * *p* < 0.05 ** *p* < 0.01, *** *p* < 0.001.

To investigate T cell polarisation, we examined the mRNA expression of lineage-defining transcription factors: Tbet (Th1), GATA3 (Th2), and Foxp3 (Tregs). T-bet, a master regulator of Th1 differentiation, governs IFN-γ expression [31], whereas GATA3 promotes Th2 responses and suppresses Th1 lineage factors [32] and Foxp3 is necessary for Treg development [33]. Infected mice exhibited low T-bet expression, which was significantly upregulated in all treated groups, most notably after ABZ and ABZ+HLE therapy (*p* < 0.001) (Figure 3A). In contrast, both GATA3 and Foxp3 were markedly elevated in infected and ABZ-only groups, while their expression levels significantly declined following HLE and combination therapy (*p* < 0.001) (Figure 3B,C), indicating a rebalancing of Th1/Th2/Treg responses.

### 2.4. Gene Expression of Breg-Associated Markers

**Figure 4:**
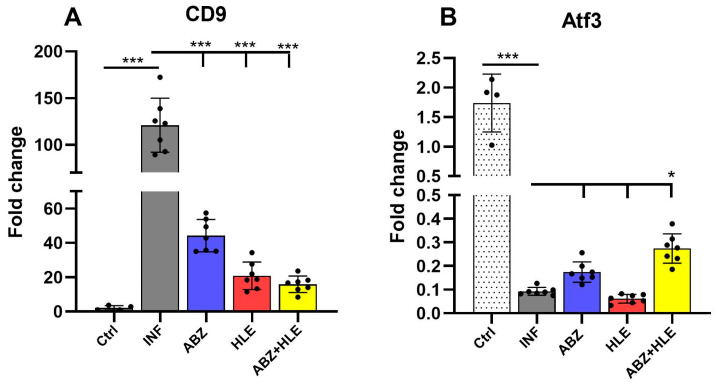
Gene expression of Breg-associated markers *Cd9* and *Atf3* in peritoneal lymphocytes. (**A**) Relative mRNA expression levels of *Cd9* and (**B**) *Atf3* were assessed by qPCR in lymphoid cell populations isolated from the peritoneal cavity of infected and treated mice. Data are shown as mean ± SEM (means of triplicate analysis) (*n* = 7); * *p* < 0.05, *** *p* < 0.001.

We analysed mRNA expression levels of Breg-associated markers *Cd9* and *Atf3* in peritoneal lymphocytes [34]. The expression of *Cd9*, a surface marker characteristic of Bregs, was significantly upregulated in infected mice but markedly reduced in all treated groups (*p* < 0.001), indicating effective suppression of the Breg phenotype by the therapies (Figure 4A). Conversely, infection with *M. vogae* led to a downregulation of *Atf3*, a transcription factor involved in cellular stress responses, suggesting stress-induced modulation of the lymphoid microenvironment. Combination therapy induced a modest increase in *Atf3* expression, although this did not correspond with the changes observed in *Cd9* expression.

### 2.5. Proliferation of ConA- and LPS-Stimulated Lymphocytes and Response to E/S Antigens

**Figure 5:**
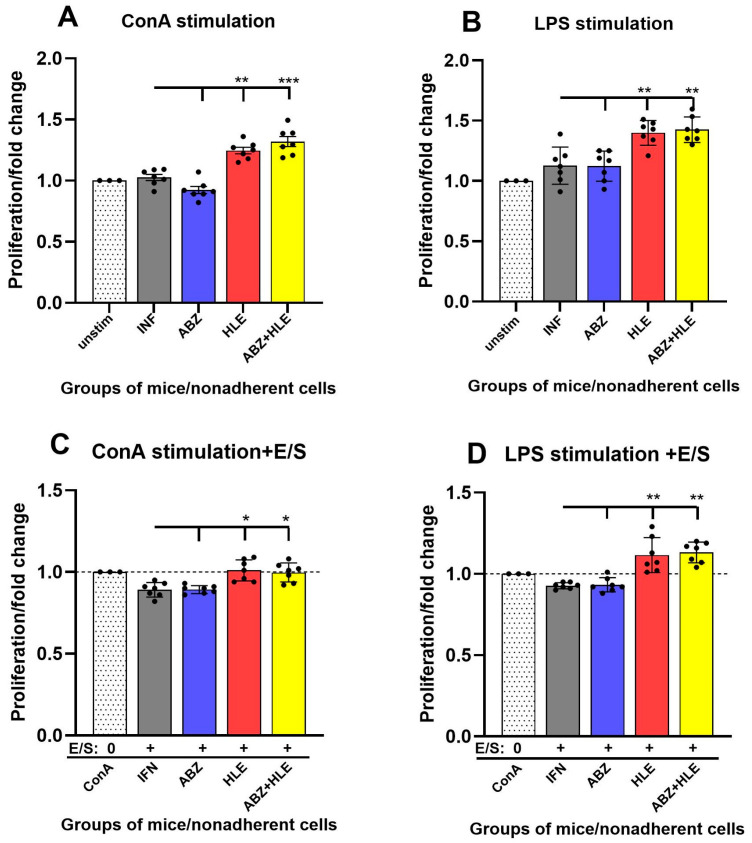
Proliferative responses of peritoneal lymphocytes to mitogenic stimulation and larval E/S antigens. BrdU incorporation assay was used to assess proliferation of non-adherent peritoneal lymphocytes after 72 h culture. (**A**) Fold change in proliferation after ConA stimulation (T cells). (**B**) Fold change after LPS stimulation (B cells). (**C**,**D**) Re-stimulation of mitogen-activated T and B cells with larval E/S products. Data are shown as mean ± SEM (means of triplicate analysis) (*n* = 7); * *p* < 0.05 ** *p* < 0.01, *** *p* < 0.001 vs. infected group.

Helminth-derived excretory/secretory (E/S) products are known to modulate host immunity to promote parasite survival [35]. To assess lymphocyte functionality, we evaluated the proliferative capacity of non-adherent peritoneal lymphocytes ex vivo using BrdU incorporation following 72-h stimulation with ConA (T cell mitogen), LPS (B cell mitogen), or re-stimulation with larval E/S antigens. Cells from infected and ABZ-treated mice exhibited significantly reduced proliferative responses to ConA, indicative of impaired T cell function. In contrast, proliferation was partially restored in the HLE and ABZ+HLE groups (*p* < 0.001) (Figure 5A). Similarly, LPS-induced B cell proliferation was low in infected and ABZ-treated groups but showed moderate recovery following HLE and combination therapies (Figure 5B). Re-stimulation with E/S antigens led to further suppression of T and B cell proliferation across all groups, with the most profound inhibition observed in the infected and ABZ-only groups. However, cells from the HLE and ABZ+HLE groups displayed a partial restoration of responsiveness despite E/S exposure (Figure 5C,D), suggesting that HLE may mitigate E/S-induced immune hyporesponsiveness through its immunomodulatory components.

### 2.6. Production of IFN-γ, IL-4, and IL-10 by Stimulated Peritoneal Lymphocytes

**Figure 6:**
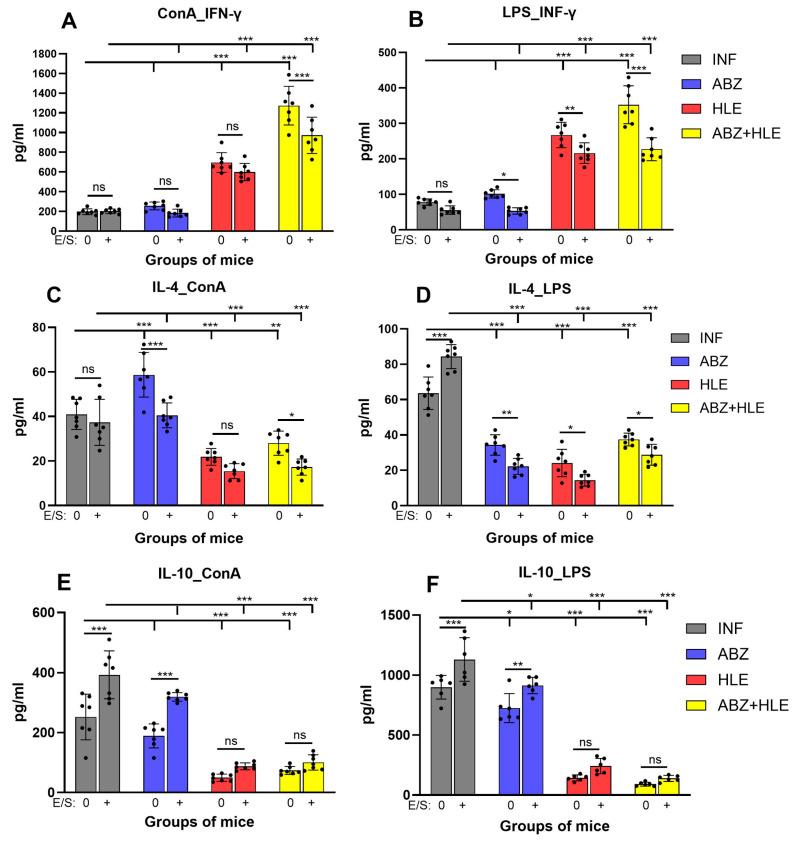
Cytokine production by stimulated peritoneal lymphocytes. Levels of IFN-γ, IL-4, and IL-10 were measured in culture supernatants of non-adherent peritoneal cells after 72 h of stimulation with ConA (T cell mitogen), LPS (B cell mitogen), or re-stimulation with *Mesocestoides vogae* E/S antigens. (**A**,**C**,**E**) Cytokine levels from ConA-stimulated cultures representing T cell responses. (**B**,**D**,**F**) Cytokine levels from LPS-stimulated cell cultures representing B cell responses. Data represent mean ± SEM (means of triplicate analysis) (*n* = 7); * *p* < 0.05 ** *p* < 0.01, *** *p* < 0.001 vs. infected group. Differences between untreated and E/S treated cells are indicated by connecting line; ns = not significant.

The polarising effects of the treatments on T and B lymphocytes were evaluated by quantifying the canonical Th1/Th2/Treg cytokines IFN-γ, IL-4, and IL-10 in culture supernatants following stimulation with ConA, LPS or re-stimulation with larval E/S antigens. IFN-γ production by ConA-stimulated T cells was low in infected and ABZ-treated mice and significantly increased in the HLE and ABZ+HLE treated groups (*p* < 0.001), consistent with elevated *T-bet* expression and proliferative activity (Figure 6A). LPS-stimulated B cells from infected and ABZ-treated mice produced only low levels of IFN-γ, whereas B cells from HLE and ABZ+HLE groups secreted significantly higher levels, though E/S antigens reduced this secretion in all groups. (Figure 6B). Compared to the infected group, IL-4 levels were significantly increased in cultures of ConA-stimulated T cells from ABZ-treated mice (*p* < 0.001) and were markedly reduced in the HLE and ABZ+HLE groups (Figure 6C). In contrast, LPS-stimulated B cells from infected mice secreted high levels of IL-4, which declined in all treated groups, suggesting differential regulatory effects of ABZ on T and B1 cells (Figure 6D). Notably, IL-4 production was also suppressed in all groups following stimulation with E/S antigens.

Production of the regulatory cytokine IL-10, is feature of regulatory Tregs and Bregs to control over-activated inflammation and maintain suppression by helminth parasites. IL-10 was elevated in cultures of stimulated T and B cells from infected mice and only partially reduced following ABZ therapy. Significant decline was detected in the cell supernatants from HLE and ABZ+HLE treated groups (Figure 6E,F). Co-culture with E/S antigens significantly elevated IL-10 levels in the infected and ABZ-treated groups, confirming the immunosuppressive role of larval products, whereas the addition of HLE partially attenuated this effect.

### 2.7. Analysis of Antibody Isotypes and Immunoreactive E/S and MvH Antigens in Peritoneal Exudates and Detection of Antibody Secreting Peritoneal Cells

Flow cytometric analysis of lymphoid subpopulations revealed elevated proportions of antibody-secreting plasma cells and plasmablasts. Therefore, we investigated the effects of the treatments on IgM and IgG antibody subclass levels in peritoneal exudates, with specificity to larval somatic (MvH) and excretory/secretory (E/S) antigens. In addition, the expression profiles of immunogenic molecules were examined by Western blotting, and the presence of isotype-specific antibody-secreting cells was demonstrated by immunocytochemical staining of lymphoid populations.

**Figure 7:**
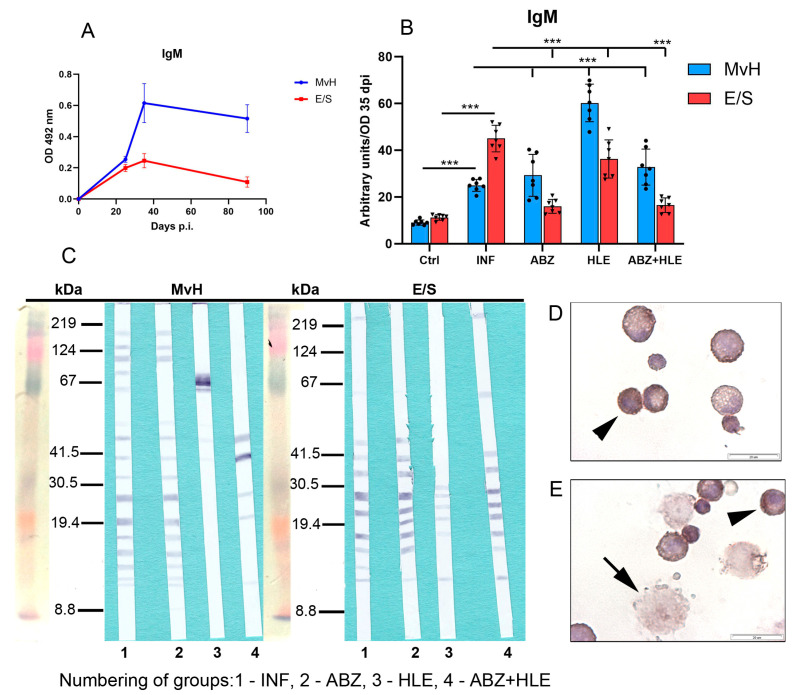
IgM antibody response and immunoreactivity to MvH and E/S antigens in peritoneal exudates of *M. vogae* infected mice. (**A**) Kinetics of IgM levels in infected untreated mice showed a peak between days 35 and 40 p.i. (**B**) IgM antibody levels specific to MvH and E/S antigens in treated groups. Data represent mean ± SEM (means of triplicate analysis) (*n* = 7); *** *p* < 0.001. (**C**) Representative images from Western blot analysis showing revealed distinct immunoreactive profiles for MvH and E/S antigens across treatment groups (1-INF, 2-ABZ, 3-HLE, 4-ABZ+HLE). (**D**,**E**) Immunocytochemical staining of non-adherent lymphoid cells with HRP-conjugated anti-IgM antibodies identified IgM-producing plasma cells (arrowhead) and large plasmablast-like cells (arrow) (scale bar = 20 µM).

Monitoring the kinetics of IgM levels in infected untreated mice revealed peak concentrations between days 35 and 40 post-infection (p.i.), followed by a gradual decline (Figure 7A). These optical density (OD) values were used to calculate arbitrary units for antibody levels across experimental groups (Figure 7B). Compared with infected controls, IgM levels specific to MvH antigens were elevated in all treated groups, with the highest increase observed following HLE therapy (*p* < 0.001). In contrast, IgM levels specific to E/S antigens were significantly reduced in the ABZ and ABZ+HLE treatment groups. Compared with infected controls, treated groups displayed altered antigen recognition profiles for MvH and E/S, with some bands absent and others newly appearing in (Figure 7C). Immunocytochemical staining of non-adherent lymphoid cells using HRP-labelled monoclonal anti-IgM antibodies revealed IgM-specific plasma cells. In addition, large, strongly stained cells, likely representing short-lived plasmablasts with irregular plasma membranes, were frequently observed (Figure 7D,E).

**Figure 8:**
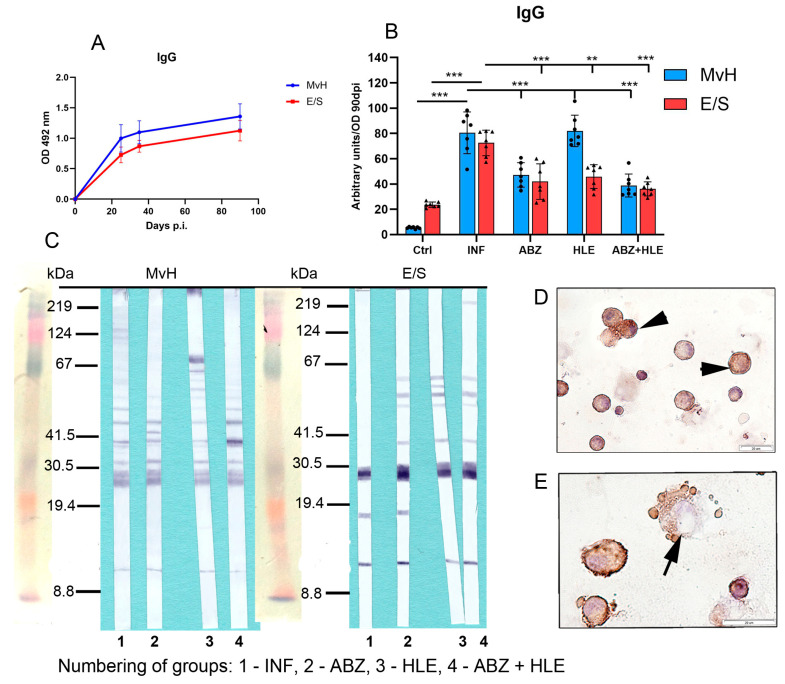
IgG antibody levels and immunoreactivity of MvH and E/S antigens in peritoneal exudates. (**A**,**B**) Total IgG antibody levels specific to MvH (**A**) and E/S. (**B**) Antigens were measured in peritoneal exudates by ELISA. Arbitrary units were calculated from OD values on day 90 p.i. Data represent mean ± SEM (means of triplicate analysis) (*n* = 7); ** *p* < 0.01, *** *p* < 0.001. (**C**) Representative images from Western blot analysis showing differential profiles of immunoreactive MvH and E/S antigens for individual groups (1-INF, 2-ABZ, 3-HLE, 4-ABZ+HLE). (**D**,**E**) Immunocytochemical detection of IgG-producing peritoneal cells showing positive staining in enlarged plasma cells (arrowhead) and a smaller number of plasmablasts (arrows); (scale bar = 20 µM).

Evaluation of total IgG antibodies revealed activation of B cell differentiation into plasma cells, as IgG titres against both antigens increased with the progression of infection. Optical density (OD) values corresponding to IgG titres on day 90 p.i., representing the chronic stage of infection, were used to calculate arbitrary units (Figure 8A). IgG antibodies specific to MvH antigens were elevated in both the infected group and HLE-treated group and significantly declined following ABZ treatment alone and in combination with HLE (*p* < 0.001), likely due to the decreased parasite burden. Similarly, IgG levels specific to E/S antigens were markedly elevated in the infected group but significantly reduced in all treated groups, most notably following combination therapy (Figure 8B), suggesting that HLE may interfere with larval secretory activity on molecular level. Western blot analysis revealed a differential expression profile of MvH-immunoreactive antigens in peritoneal exudates among all experimental groups (Figure 8C), which differed substantially from the profiles observed after probing E/S-specific membranes. In all groups, IgG antibodies prominently recognised a dominant ~27 kDa antigen, while exudates from treated mice also reacted with additional antigens in the 35–67 kDa molecular weight range. Immunocytochemical staining confirmed IgG immunoreactivity in enlarged plasma cells and, to a lesser extent, in plasmablasts (Figure 8D,E).

**Figure 9:**
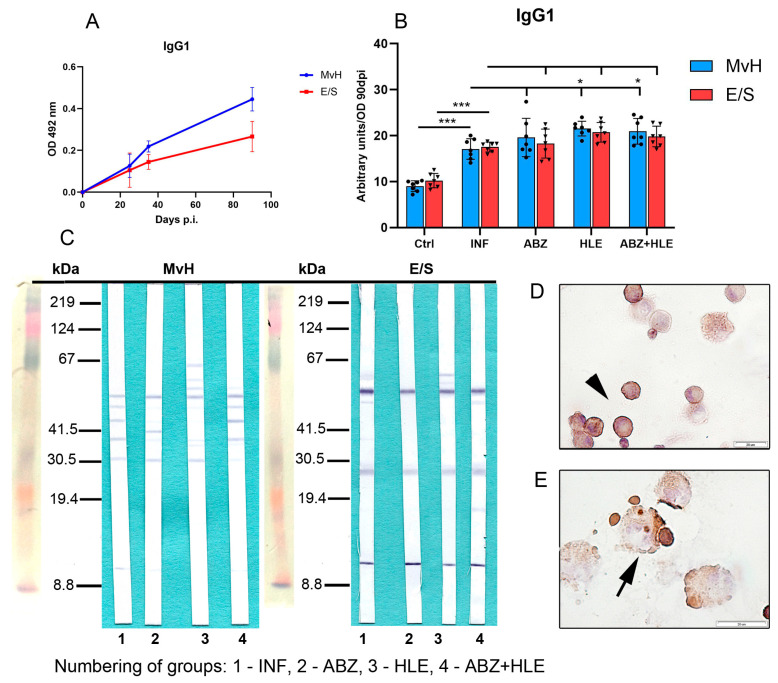
IgG1 antibody levels and antigen recognition patterns in peritoneal exudates. (**A**) IgG1 levels specific to MvH and E/S antigens on day 90 p.i., expressed in arbitrary units derived from OD values. (**B**) IgG1 levels to MvH remained unchanged across groups, while E/S-specific IgG1 increased post-treatment. Data represent mean ± SEM (means of triplicate analysis) (*n* = 7); * *p* < 0.05, *** *p* < 0.001. (**C**) Western blot profiles showing differential IgG1 reactivity to MvH and E/S antigen for individual groups (1-INF, 2-ABZ, 3-HLE, 4-ABZ+HLE). (**D**,**E**) IgG1-producing plasma cells (arrowhad) and plasmablasts (arrow) detected by immunocytochemistry (scale bar = 20 µM).

The proportions of antibody subclasses vary depending on the prevailing cytokine environment [36]. IgG1 production is typically induced by IL-4 [37]. OD values for IgG1 specific to MvH and E/S antigens measured at day 90 p.i. were used to calculate arbitrary units representing IgG1 levels in peritoneal exudates (Figure 9A). Interestingly, IgG1 responses showed a different pattern from total IgG. MvH antigens elicited a weaker IgG1 response than E/S antigens, with no significant differences in IgG1 levels to MvH between infected and treated groups (Figure 9B). However, IgG1 levels to E/S antigens increased following therapy, particularly in the HLE group (*p* < 0.05). Western blotting revealed weak reactivity with MvH antigens (30–67 kDa) in all groups, with altered band patterns in treated animals. In contrast, IgG1 antibodies strongly recognised three dominant E/S antigens across all groups, with additional bands emerging after HLE or combined treatment (Figure 9C). Immunocytochemical staining confirmed IgG1 secretion by plasma cells and plasmablasts (Figure 9D,E).

**Figure 10:**
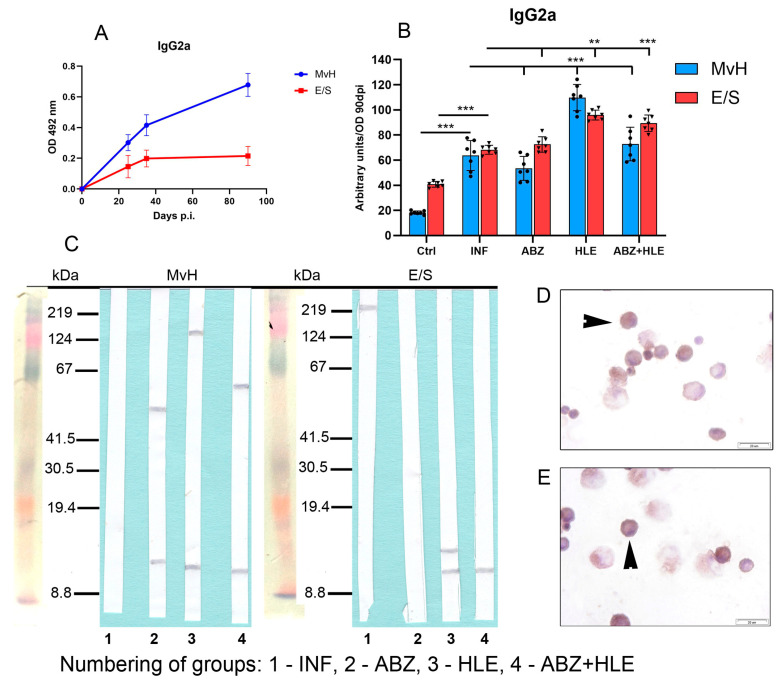
IgG2a antibody response and antigen recognition in peritoneal exudates and cell staining. (**A**) ELISA quantification of IgG2a levels specific to MvH and E/S antigens. (**B**) IgG2a levels in exudates from infected and treated groups of mice. Data represent mean ± SEM (means of triplicate analysis) (*n* = 7); ** *p* < 0.01, *** *p* < 0.001. (**C**) Western blotting analysis for individual groups (1-INF, 2-ABZ, 3-HLE, 4-ABZ+HLE) revealed weak IgG2a reactivity; low-MW bands (~12 kDa) were visible in treated groups. (**D**,**E**) Immunocytochemical localisation of IgG2a-secreting plasma cells (arrowhead) (scale bar = 20 µM).

Previous studies have shown that Th1 cytokines, especially IFN-γ, promote IgG2a and IgG3 production, while TGF-β induces class-switching to IgG2b [37]. We assessed IgG2a and IgG2b levels specific to MvH and E/S antigens, using OD values from day 90 p.i. to calculate arbitrary units (Figure 10A). Overall, IgG2a concentrations were lower than IgG1 and IgM. While ABZ therapy did not enhance IgG2a levels, significantly higher concentrations were observed in mice treated with HLE and combination therapy (Figure 10B), consistent with observed increased IFN-γ secretion ex vivo. Western blotting revealed weak IgG2a reactivity with MvH in infected mice, with different antigen profiles in treated groups. For E/S antigens, only faint bands were visible, with low-MW antigens (~12 kDa) detected in exudates obtained after HLE and combination therapy (Figure 10C). IgG2a-secreting cells were localised to smaller plasma cells (Figure 10D,E).

**Figure 11:**
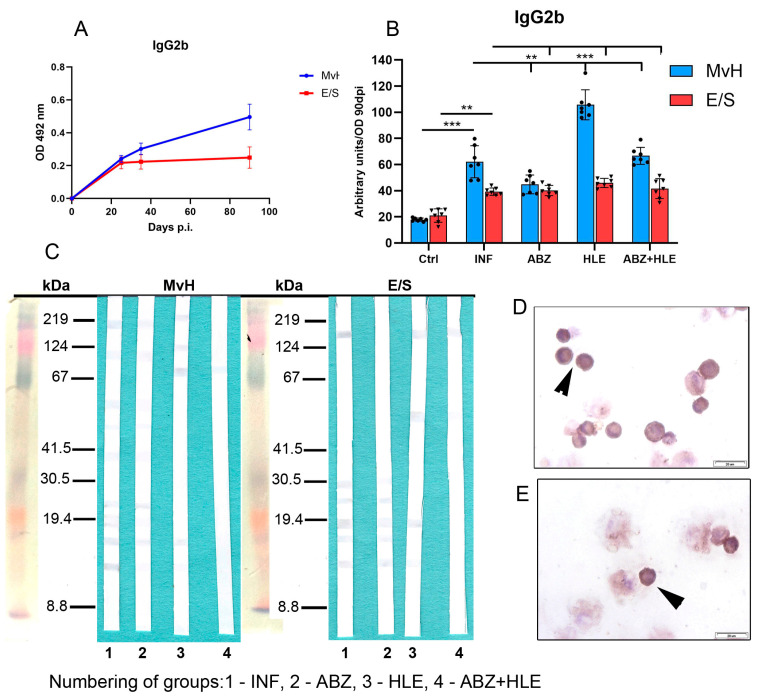
IgG2b antibody response, antigen recognition in peritoneal exudates and antibody secreting cells. (**A**) IgG2b levels expressed as arbitrary units at day 90 p.i. (**B**) Effect of infection and treatments on MvH and E/S-specific IgG2b levels. Data represent mean ± SEM (means of triplicate analysis) (*n* = 7); ** *p* < 0.01, *** *p* < 0.001. (**C**) Western blot detection of MvH and E/S antigens recognised by IgG2b in individual groups (1-INF, 2-ABZ, 3-HLE, 4-ABZ+HLE). (**D**,**E**) Representative images of IgG2b-secreting plasma cells (arrowhead), (scale bar = 20 µM).

Infection induced higher IgG2b responses to MvH than to E/S antigens. Surprisingly, a significant increase in MvH-specific IgG2b was detected in the HLE group (*p* < 0.001), while E/S-specific IgG2b levels were not significantly altered by any treatment (Figure 11B). Correspondingly, more immunogenic MvH antigens were detected in exudates from infected mice, whereas responses were markedly diminished following ABZ+HLE therapy. Similarly, E/S-specific IgG2b reactivity was weak across all groups, with fewer bands observed in HLE and combination-treated mice than in infected controls (Figure 11C). Smaller IgG2b-expressing plasma cells were identified by a weak immunocytochemical staining (Figure 11D,E).

### 2.8. Efficacy of Treatments on Larval Burden and mRNA Levels of the Regulatory 14-3-3 Gene

**Figure 12:**
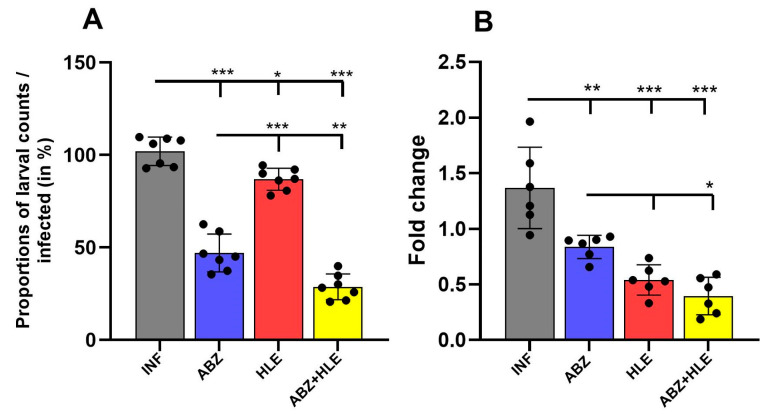
Larvicidal efficacy of ABZ, HLE, and combination therapy against *Mesocestoides vogae* infection. (**A**) Total larval burden in the peritoneal cavity of mice following treatment with albendazole (ABZ), human leukocyte extract (HLE), or their combination (ABZ+HLE). (**B**) Relative mRNA expression of the larval *14-3-3* gene, a marker of cellular proliferation and development showing significant downregulation in all treated groups to the infected control group. Data are expressed as means ± SEM (means of triplicate analysis) (*n* = 7); * *p* < 0.05 ** *p* < 0.01, *** *p* < 0.001.

The larvicidal effects of ABZ, HLE, and their combination are shown in Figure 12A. In comparison with infected mice, the greatest reduction in larval burden in comparison with counts in infected mice (%) was observed in mice treated with the combination of ABZ and HLE, and less effective was ABZ monotherapy (*p* < 0.001). Intraperitoneal administration of HLE alone led to a modest reduction of larval counts and efficacy, indicating a synergistic effect. To determine whether the reduction in larval burden was associated with interference in larval proliferation or development, we examined the expression of the *14-3-3* gene. This gene plays a critical role in cell cycle regulation and general physiology in a wide range of eukaryotic organisms, including helminths [38]. We quantified 14-3-3 mRNA expression in larvae isolated from treated and untreated mice. Compared to larvae from the infected control group, expression of *14-3-3* was significantly downregulated in all treated groups, with the most pronounced suppression observed in ABZ+HLE treated group, followed by HLE alone and then ABZ monotherapy (Figure 12B). Results suggest that both HLE and the combination treatment impair larval proliferation, likely through disruption of regulatory pathways essential for parasite development.

## 3. Discussion

In the infected peritoneal cavity, *M. vogae* induces a marked increase in myeloid-derived suppressor cells (MDSCs), comprising predominantly M2 macrophages and eosinophils, at the expense of lymphoid populations [25,39,40], along with elevated concentrations of the Th2/Treg-associated cytokines IL-4, IL-10 and transforming growth factor-β (TGF-β). This permissive immune environment, typically elicited by helminths, facilitates parasite survival and proliferation while limiting host tissue pathology. Ideally, an effective treatment would not only eliminate the parasite but also induce host-protective immune responses that enhance the efficacy of chemotherapy and reduce the adverse effects of drugs.

In the present study, we examined the effects of ABZ, the immunomodulatory product HLE, and their combination on non-adherent lymphoid cell populations in the peritoneal cavity of BALB/c mice, using experimental conditions identical to our previous investigation focused on myeloid populations [39]. The greatest reduction in larval burden was observed following combined ABZ+HLE therapy, while HLE monotherapy resulted in a modest yet significant decrease, supporting its role as a beneficial adjuvant.

In our study, flow cytometric analysis of non-adherent cells revealed that CD3^+^ T lymphocytes and CD19^+^ B lymphocytes were present at lower frequencies compared with antibody-secreting CD138^+^ plasmablasts and various plasma cell stages. Immune modulation towards a Treg phenotype, coupled with suppression of Th1 responses, is a common feature of helminth infections, documented, for *E. multilocularis* [30], *Taenia crassiceps* [41], and *M. vogae* [25,42]. In our study, a significant increase in CD3^+^ T cells and a decrease in CD19^+^ B cells were observed following HLE and combined ABZ+HLE therapy compared with untreated infected controls. CD3^+high^ cells predominated after HLE and combined therapy, whereas their frequency slightly decreased following ABZ given alone. As CD3 is a multimeric protein complex that associates with the T cell receptor (TCR) and is essential for T cell lineage commitment [27], these results suggest that components of HLE may modulate this process.

The peritoneal cavity resides a distinct subset of B cells, known as CD19^+^ B1 cells, which differ from conventional B2 cells in phenotype, function, and self-renewal capacity [43,44]. Upon antigen encounter, parasite-induced B cells can differentiate into short-lived plasmablasts or undergo clonal expansion into high-affinity antibody-producing plasma cells and memory B cells. In our study, a high frequency of plasmablasts/plasma cells was noted following infection and ABZ therapy, whereas a significant decline was recorded after combined treatment, correlating with the greatest reduction in parasite burden. To further investigate the impact of therapy on regulatory lymphocyte subtypes, we examined IL-10-secreting CD3^+^ Treg and CD19^+^/IL-10 Breg cells using intracellular staining. Regulatory B cells (Bregs) are known to suppress various immune cells, including T cells, dendritic cells and monocytes, partly by promoting regulatory T cells (Tregs) [45]. IL-10-producing T cell frequencies were reduced following HLE and combined treatment, but not after ABZ administered alone. In contrast, IL-10-producing B1 cells were significantly reduced in all treated groups. Collectively, these data indicate that HLE, particularly in combination with ABZ, effectively reversed the infection-driven dominance of Treg/Breg populations. This finding aligns with our previous study, which showed that HLE and combination therapy shifted peritoneal macrophage polarisation from M2 to M1, mediated via STAT-1/IFN-γ signalling pathway activation [39]. M1 versus M2 polarisation is driven by IFN-γ and IL-4, respectively, with M2 macrophages being prominent producers of IL-10 [46].

To date, limited information is available on the effects of HLE on lymphocyte phenotypes and cytokine profiles during flatworm infections. In a related murine model of *E. multilocularis* infection, combination therapy with ABZ and a swine-derived dialysable leukocyte extract (Imunor, ImunomedicA, Ltd., Ústí nad Labem, Czech Republic) resulted in an expanded splenic CD4^+^ T cell population with elevated IFN-γ production [47]. In the same model, HLE therapy (Immodin, SevaPharma Ltd., Prague, Czech Republic) downregulated IL-4 and TGF-β production by ConA-stimulated lymphocytes ex vivo, alongside suppression of *GATA3* and *Foxp3* gene expression [6]. A human leukocyte extract (Transferon^®^, Instituto Politecnico Nacional, Mexico City, Mexico) promoted the differentiation of human haematopoietic stem/progenitor cells into CD11c^+^ NK-like cells and stimulated the proliferation of bone marrow CD34^+^ cells [48], The immunomodulatory effects of ABZ on the immune system have also been consistently monitored. For example, in the liver biopsies from patients infected with *E. multilocularis* and treated with high doses of ABZ, lesions surrounding the parasitic cysts exhibited significantly increased infiltration of CD4^+^ T cells, CD20^+^ B cells, and CD38^+^ plasma cells, indicative of an activated adaptive immune response [49].

We further explored the association between treatment-induced changes in lymphocyte phenotypes and the expression of key transcription factors orchestrating T cell polarisation. Compared to infected mice, ABZ treatment significantly upregulated *T-bet* and *GATA3*, and partially downregulated *Foxp3*, demonstrating a mixed polarisation profile. However, HLE and especially ABZ+HLE therapy induced a more balanced Th1/Th2 response, characterised by strong *T-bet* activation, moderate *GATA3* downregulation, and more pronounced *Foxp3* suppression. This was consistent with the lower proportions of IL-10-producing CD3^+^ cells observed in these groups. Regulatory T cells (Tregs; CD4^+^CD25^+^Foxp3^+^) mediate their suppressive effects on adaptive immunity by inhibiting T cell proliferation, downregulating pro-inflammatory cytokines such as IFN-γ and TNF-α, and promoting the secretion of TGF-β and IL-10, which also induce M2 macrophage polarisation [50]. Increased CD4^+^CD25^+^Foxp3^+^ Tregs have also been documented in the peritoneal cavity of mice intraperitoneally infected with *E. multilocularis*, and their depletion improved infection control [51]. Our results suggest that ABZ+HLE therapy effectively restricted Treg expansion while supporting Th1-polarised, anti-parasitic immunity.

B cells, including the B1 subset, serve as an important source of cytokines and as precursors of plasma cells, playing pivotal roles in coordinating interactions among macrophages, dendritic cells, and T cells [52]. In another flatworm infection, schistosomiasis, *CD9* has recently been identified as a key surface marker for murine IL-10^+^ CD19^+^ Bregs involved in infection progression [53]. In our study, expression of *Cd9* was strongly upregulated in B1 cells from infected mice and significantly downregulated in all treated groups, including the ABZ treated mice. This was not entirely consistent with the pattern of *Foxp3* expression, suggesting that ABZ may have exerted a more pronounced molecular effect on B cells than on peritoneal T cells. We also found that in comparison with control larval infection strongly downregulated *Atf3* gene expression, another transcription factor associated with Breg function, immune regulation, and homeostasis [34,54] and therapies only moderately modulated mRNA levels. 

Attenuated T cell proliferation is a hallmark of helminth-induced immunosuppression, often directed by E/S antigens. To assess whether changes in lymphocyte phenotype were translated into altered functional capacity, we stimulated peritoneal T and B1 cells with ConA or LPS and measured proliferation. T cells from infected and ABZ-treated mice exhibited poor proliferative responses to ConA, which were partially restored following HLE treatment and markedly improved by ABZ+HLE therapy. ConA, lectin, which binds mannose residues on T cell membranes and is internalised to mitochondria [55], induces proliferation and cytokine release. Thus, the enhanced proliferation suggests a shift towards a more functional T cell pool. The T cell proliferation after re-stimulation with parasite products was hardly detectable in all groups, indicating a state of functional anergy, a known helminth-induced immune evasion mechanism that preserves lymphocyte viability while impairing effector function [56]. Helminth-derived molecules are also known to inhibit TLR4 signalling in myeloid cells; for instance, glycomolecules from *E. granulosus* were shown to impair TLR4-mediated activation in dendritic cells [57]. In our study, LPS-stimulated B1 cell proliferation was poor in infected mice but was partially restored by HLE and combination therapy. This suggests that HLE may counteract the inhibitory effects of E/S products on TLR4 signaling in B1 cells.

Cytokine production in response to mitogen stimulation reflected these changes in functional responsiveness. In our study, IFN-γ secretion increased significantly in HLE and combination-treated groups after ConA or LPS stimulation, while IL-4 and IL-10 levels declined. This shift away from Th2/Treg cytokines further confirms reversal of helminth-induced immunosuppression. Notably, ABZ alone was less effective in restoring IFN-γ production and dampening IL-10 secretion. There is little known regarding the effects of oral ABZ therapy on peritoneal lymphoid population during cestode infections in mice. Clinical data on the effects of ABZ in echinococcosis patients revealed significant reductions in IL-10 and partial reductions in IL-4 in serum that was associated with the successful therapy [58,59]. In *M. vogae* infected mice, the role of IL-4 appears complex. C57BL/6 mice are more resistant to this infection than BALB/c and IL-4-deficient (IL4^−^/^−^) C57BL/6 mice exhibited increased parasite burdens, heightened IFN-γ and TNF-α production, and a shift from IgG1 to IgG2a antibodies [40,60]. These findings suggest that while Th1 responses are important for parasite control, as demonstrated by IFN-γ’s larvicidal effects [61], a balanced Th1/Th2 profile may be more effective. Our data support this conclusion, with ABZ+HLE therapy producing the most balanced and protective immune profile.

Antibodies are essential effectors of adaptive immunity, with isotype switching governed by the cytokine milieu. In mice, elevated secretion of IL-4 promotes IgG1 and IgE, while IFN-γ induces IgG2a and IgG3 and inhibits Th2-associated isotypes [37]. In mouse *M. corti* infection, larval antigens selectively induced IgM and IgG1 in serum [62], and similar subclass patterns were found across four different helminth infections [63], including *M. corti*. We observed relatively strong peritoneal antibody responses dominated by IgM and IgG and antigen-specific variations in isotype reactivity. Treatments significantly modulated these responses. ABZ+HLE therapy resulted in the greatest reduction of total IgG and IgM, which corresponded with the lowest CD138^+^ plasma cell counts and larval burden. Interestingly, IgG1, the isotype dominant in systemic responses to *M. corti*, did not prevail in the peritoneal cavity, what may reflect reduced IL-4 levels in this microenvironment. Notably, elevated IFN-γ correlated with increased IgG2a levels after HLE and combination therapy, indicating a Th1-driven class switch. These differential antibody profiles highlight the complex interplay between host immunity and parasite antigens, and the ability of immunomodulatory therapy to redirect these responses. Proteomic analysis of an identical HLE product, formerly marketed as Immodin, revealed the presence of 48 unique proteins/polypeptides, classified into three functional groups: molecules regulating immune responses, proteins involved in inflammatory processes and tissue repair, and proteins regulating cell growth [64].

Finally, we found a correlation of drug efficacy on larval biology by measuring expression of the *14-3-3* gene, a cell cycle regulator in eukaryotic organisms, including helminths. Expression was significantly downregulated following therapy, especially in the ABZ+HLE group. Surprisingly, HLE alone also reduced gene expression, potentially explaining its modest larvicidal activity. Similar correlations between *14-3-3* downregulation and parasite mass reduction were observed in *E. multilocularis* infected mice [65]. We suppose that suppressed *14-3-3* mRNA expression may not fully account for the inhibition of all larval pro-survival adaptation mechanisms. This could explain the relatively modest reduction in larval burden compared to the effect of ABZ, which acts directly through inhibition of tubulin polymerisation. A deeper investigation of this phenomenon, along with studies on the safety and risks of this adjuvant, could support future research and the development of innovative therapeutic strategies against parasitic diseases.

In summary, our study showed that co-administration of HLE with ABZ significantly enhanced the drug’s larvicidal efficacy, likely through synergistic immunomodulatory effects on T and B lymphocyte subsets and by suppressing expression of the parasite’s *14-3-3* gene. While ABZ alone showed limited capacity to counteract larval E/S-induced T cell suppression, it partially reduced IL-10-producing CD9^+^ Bregs and inhibited their secretion of IL-10 following LPS stimulation. However, upregulation of Th1/Th2/Treg transcription factors under ABZ monotherapy was not mirrored by reduced IL-10 production in T cells, indicating ongoing functional hyporesponsiveness. In contrast, HLE markedly improved T cell responsiveness, as evidenced by enhanced Th1 proliferation, increased *T-bet* expression, and restoration of IFN-γ production. These effects were accompanied by augmented IgG2a antibody responses and suppression of Treg- and Breg-associated markers. Downregulation of *GATA3* and reduced IL-4 production further suggested that HLE rebalanced the local Th1/Th2 response in favour of parasite clearance.

## 4. Materials and Methods

### 4.1. Albendazole and HLE

Albendazole (ABZ), used as the standard anthelmintic, was purchased from Sigma-Aldrich (St. Louis, MO, USA). For oral administration, ABZ was suspended in 1.0% cremophor oil in 0.9% sterile NaCl and stored at 4 °C. Human dialysable leukocyte extract (HLE) used in our study was manufactured by Aumed, Ltd. (Prague, Czech Republic), a GMP-certified pharmaceutical company, and supplied exclusively for research purposes. The preparation is registered in the European Union under International Publication No. WO 2021/249581 A1 and patented in the Czech Republic by the Industrial Property Office (Patent No. 34517). According to the manufacturer’s protocol, HLE is a soluble extract of peripheral blood mononuclear cells (PBMCs) obtained from over 150 healthy donors, which were screened at certified transfusion centres and tested negative for HIV 1/2, HCV, HBsAg, and syphilis. The extract contains a complex mixture of proteins and other molecules with a molecular weight below 10 kDa. Each batch was prepared by pooling leukocytes from donors, followed by disintegration using six cycles of freezing at a temperature below −50 °C and thawing at a temperature above +30 °C. The resulting homogenate was concentrated by dialysis to a defined protein concentration, and the low molecular weight fraction was isolated using 10 kDa ultrafiltration membranes and further concentrated by ultrafiltration. The final product was pasteurised and quality-controlled for sterility, endotoxins, and biological activity using proliferation assays on the Jurkat T cell line.

### 4.2. Infection and Experimental Design

Infections with *Mesocestoides vogae* (syn. *M. corti*) larvae were maintained through serial passage in ICR mice housed under pathogen-free conditions at the animal facility of the Institute of Parasitology. In the experiment, we used 7-week-old male BALB/c mice, bred at the accredited animal facilities of the Institute of Parasitology of the Slovak Academy of Sciences under pathogen-free conditions. Mice strain was originally purchased from Velaz (Prague, Czech Republic). Mice were bred under standard laboratory conditions (12 h light/dark cycle, 22 ± 2 °C, standard chow diet). Four healthy mice served as uninfected controls (Ctrl), and 28 mice were orally infected with 60–65 tetrathyridia isolated from the peritoneal cavity of ICR mouse. Infected mice were randomly allocated into four groups (*n* = 7 per group): INF—infected, untreated control; ABZ—infected, treated with albendazole; HLE—infected, treated with human leukocyte extract (HLE); and ABZ+HLE—infected, treated with both ABZ and HLE. Treatments were administered daily for 11 consecutive days, from day 15 to day 25 post-infection (p.i.). ABZ was administered orally by gavage at a dose of 10 mg/kg body weight, and HLE was delivered intraperitoneally at a dose of 0.2 mL (equivalent to an extract derived from 1 × 10^9^ leukocytes). On day 26 p.i., after termination of therapy, mice were subjected to mild anesthesia in a CO_2_ atmosphere and were then euthanised by cervical dislocation. Peritoneal exudates, peritoneal exudate cells (PECs), and larvae were subsequently collected for further analyses.

### 4.3. Isolation of Peritoneal Non-Adherent Cell Populations and Exudates

Peritoneal exudates were aseptically collected from mice by injecting 1 mL of sterile PBS. Following isolation of larvae and peritoneal cells, exudates were stored at −80 °C. Peritoneal cells were obtained via a second lavage with 5 mL of RPMI medium (Biochrom, Berlin, Germany) supplemented with 2 mM stable glutamine, 10% heat-inactivated foetal bovine serum (Biochrom, Berlin, Germany), 100 U/mL penicillin, 100 μg/mL streptomycin, 10 μg/mL gentamicin, and 2.5 μg/mL amphotericin B (complete medium, CM; all from Sigma-Aldrich, St. Louis, MO, USA). Cells from both lavages were pooled. Peritoneal exudate cells (PECs) were resuspended in CM, seeded into culture flasks, and incubated for 18 h to separate non-adherent lymphoid cells from adherent macrophages and eosinophils. Non-adherent cells were washed and counted using the trypan blue exclusion method (Sigma-Aldrich, St. Luis, MO, USA). Cell aliquots (2 × 10^6^ cells/mouse) were preserved in RiboZol reagent (VWR Chemicals, Radnor, PA, USA) and stored at −80 °C for RNA extraction. Remaining cells were subjected to flow cytometry, qPCR, proliferation assays, and immunocytochemistry. Exudates were used for antibody detection and immunoblotting.

### 4.4. Flow Cytometric Analysis

Non-adherent peritoneal cells (0.3 × 10^6^ cells/50 μL) were stained with monoclonal anti-mouse antibodies: CD45.2-PE-Cy7 (clone C.104, eBioscience, San Diego, CA, USA), CD3e-FITC (clone 145-2C11, Invitrogen, Waltham, MA, USA), CD19-PE (clone 1D3, eBioscience, San Diego, CA, USA), and CD138-APC (clone 300506, Invitrogen, Waltham, MA, USA). Other cell samples were stained with anti-mouse antibodies: CD3-PE-Cy7 (clone 17A2), CD4-FITC (clone GK 1.5), and CD8-PE (clone C:53-6.7); all from eBioscience, San Diego, CA, USA). Cells were incubated for 30 min at room temperature in the dark, washed, and analysed using a FACS Canto analyser (BD Biosciences, Franklin Lakes, NJ, USA). For intracellular cytokine detection, cells were permeabilised using the BD Cytofix/Cytoperm Kit (BD Life Sciences, Franklin Lakes, NJ, USA), then stained with anti-IL-10-PE (clone J4S5-16E3, SONY Biotechnology, San Jose, CA, USA) in combination with anti-CD3e-FITC or CD19-APC-Cy7 (clone MB 19-1), both from Invitrogen (Waltham, MA, USA). Data were analysed using FACS Diva software version 9.0 and represent mean ± standard deviation, (SD), (*n* = 7).

### 4.5. Cell Proliferation Assay

Cell proliferation was assessed using the BrdU Cell Proliferation ELISA Kit (Roche, Basel, Switzerland). Non-adherent cells (1 × 10^6^/mL, 200 μL/well) were cultured in triplicates in 96-well flat-bottom plates type Falcon, (Corning, New York, NY, USA), either unstimulated or stimulated with concanavalin A (ConA, 3 μg/mL), ConA + E/S antigens (10 μg/well), lipopolysaccharide (LPS, 1 μg/mL), or LPS + E/S for 72 h at 37 °C in 5% CO_2_. BrdU (5 μM) was added for the final 22 h. Proliferation was expressed as the fold change of absorbance in stimulated vs. unstimulated cells or as the ratio of absorbance of mitogen-stimulated cells to OD values for mitogen+E/S. Tests were performed in triplicate for each cell sample and data shown in graphs are mean ± standard error of the mean (SEM) (*n* = 7).

### 4.6. RNA Isolation and Real-Time qPCR

Total RNA was extracted from 3 × 10^6^ non-adherent cells using TRIzol reagent (Invitrogen, Waltham, MA, USA). RNA quality and quantity were determined using a NanoSpectrophotometer (AstraGene, Cambridge, UK). cDNA was synthesised from 3 μg of RNA using ReverseAid H Minus M-MuLV Reverse Transcriptase and oligo dT primers (Thermo Fisher Scientific, Waltham, MA, USA). The cDNA from each sample was used as a template for quantitative RT-PCR (qPCR) analysis using SYBR Green Mix (Bio-Rad, Hercules, CA, USA) in 20 μL reactions with 2 μL cDNA gene-specific primers (10 pM each) run for 39 cycles. Transcriptional profiles of following genes with published sequences were analysed: *T-bet* [66], *GATA3* [67], *Foxp3* [68], *Atf3* [69], *CD9* [70] and housekeeping gene *GAPDH* [71]. Primer sequences are listed in Table S1 in Supplementary Materials. Relative gene expression was calculated using the 2^−∆∆Ct^ method [72], using normalised Ct values from healthy mice as the calibrator. RNA from isolated larvae was processed similarly, with expression of *14-3-3* gene normalised to α-smooth actin reference gene. Each reaction was run in triplicates and data shown in graphs are mean ± standard error of the mean (SEM) (*n* = 7).

### 4.7. Preparation of Excretory/Secretory (E/S) and Larval Homogenate Antigens

Tetrathyridia of *M. vogae* were harvested from ICR mice under aseptic conditions and washed extensively with LPS-free DPBS (Sigma-Aldrich, St. Luis, MO, USA). Surface host cells were removed by 48 h incubation in RPMI, without FCS, supplemented with antibiotics (culture medium) under standard culture conditions. Larvae were then intensively washed, transferred to fresh medium, and incubated for 7 days in culture medium. The culture supernatant after the first 24 h of culture was discarded. From day 2, supernatants were collected daily, filtered in 0.22 μm filters (Sarstedt, Numbrecht, Germany), concentrated, and buffer-exchanged into endotoxin free-DPBS using 3 kDa columns (Merck Millipore, Merck KGaA, Darmstadt, Germany). Larval viability was confirmed by motility and morphology. A portion of viable larvae was used for preparation of mixture of somatic antigens (*Mesocestoides vogae* homogenate, MvH). Larvae were sonicated in DPBS (30 s, 25% watt on ice), centrifuged at 10,000× *g*, and the supernatant (MvH) was collected. Protein content was quantified using the Bradford assay (Bio-Rad) and BSA as standard. The same batch of E/S and MvH was used throughout the study.

### 4.8. Detection of Antibodies to E/S and MvH Antigens

Enzyme-linked immunosorbent assay (ELISA) was used to quantify levels of total IgG, IgM, IgG1, IgG2a, and IgG2b antibodies against E/S and MvH antigens in peritoneal exudates. Plates (Nunc Maxisorp, Roskilde, Denmark) were coated with 2.5 μg/mL E/S or 1.5 μg/mL MvH antigens in carbonate–bicarbonate buffer (pH 9.6) overnight at 4 °C. After blocking with 10% FBS, 0.05% Tween-20 in PBS for 1 h, plates were incubated with diluted exudates (1:50) for 1 h at 37 °C. After washing, the secondary HRP-conjugated goat anti-mouse antibodies were used at the following dilutions: anti-IgG1 (1:2000), anti-mouse IgG (1:4000), anti-IgM (1:2000), anti-IgG2a (1:2000), and anti-IgG2b (1:2000), all from Abcam (Cambridge, United Kingdom). Signals were developed using o-phenylenediamine substrate in citrate buffer (pH 4.5) after addition of H_2_O_2_, and the reaction was stopped with 2 M H_2_SO_4_. Absorbance was measured at 492 nm using Multiskan FC reader (Thermo Fisher Scientific, Waltham, MA, USA). Results were expressed in arbitrary units (AU) relative to OD for calibrator, representing exudates from chronically infected mice (*n* = 3). Each sample was examined in triplicates and data shown in graphs are mean ± standard error of the mean (SEM) (*n* = 7).

### 4.9. Immunoblot Analysis

Expression of immunogenic larval MvH and E/S antigens present in the peritoneal exudates was performed by Western blotting to examine immunoreactivity with antibodies IgG, IgM, IgG1, IgG2a and IgG2b. Proteins (10 μg/lane) were denatured at 95 °C for 5 min, resolved by 12% SDS-PAGE, and transferred onto PVDF membranes (0.45 μm, Merck Millipore, Merck KGaA, Darmstadt, Germany) using a Mini Trans-Blot Electrophoretic Transfer Cell (Bio-Rad, Hercules, CA, USA) including protein Kaleidoscope prestained standard, cat. No.161-0324). Nonspecific binding was blocked upon incubation of strips in 5% fat-free milk in PBS for 2 h. Membranes with blotted E/S or MvH antigens were then incubated with pooled exudates (*n* = /group) at 1:20 dilution (IgG, IgG1, IgM) or 1:10 (IgG2a, IgG2b) in 3% fat-free milk overnight at 10 °C using IKA Roller 6 (Biosan, Riga, Latvia). After washing, HRP-conjugated goat anti-mouse antibodies were applied at the dilutions of 1:500 for IgG, IgG1, IgM and 1:200 for IgG2a, IgG2b (Abcam). Finally, immunoreactive bands were visualised using 4-chloronaphthol with H_2_O_2_. Figures show representative immunoblots.

### 4.10. Immunocytochemical Staining

Non-adherent cells were fixed in 4% paraformaldehyde and processed for intracellular immunodetection of antibody subclasses. Following permeabilisation with 0.5% Triton-X100/PBS, slides were treated with 0.3% H_2_O_2_ for 20 min to block endogenous peroxidase and nonspecific bindings was blocked with 5% goat serum/PBS. Slides were then incubated in HRP-conjugated antibodies to IgG, IgM, IgG1, IgG2a, and IgG2b (Abcam, 1:100) overnight at room temperature. Following washing, and incubation with substrate (0.4% DAB in Tris-NaCl) for 10 min, slides were counterstained with Gill’s haematoxylin, differentiated in Scott’s tap water (pH 8.2), mounted, and analysed microscopically using Olympus BX51 (Hachioji, Tokyo, Japan).

### 4.11. Larval Burden and Therapeutic Efficacy

Larvae were recovered from the peritoneal cavity, washed, and resuspended in an appropriate volume of 0.1% agar to prevent sedimentation. The suspension was thoroughly mixed, and 0.5 mL was randomly taken, placed on a glass slide, and the larvae were counted under a light microscope. For each sample, counts were performed in triplicate. The number of larvae per ml was calculated and then extrapolated to the total volume of the larval suspension in agar. Approximately 50 mg of larvae per mouse were preserved in TRIzol (Invitrogen) at −80 °C for RNA extraction. Therapeutic effect was expressed as the proportions of larval numbers/mouse in treated groups calculated as percentage from infected untreated group, where the mean number (*n* = 7) represented 100%. Data represent mean ± stand error of the mean (SEM).

### 4.12. Statistical Analysis

Depending on the assay used, analyses were performed in duplicates or triplicates and the mean was calculated and was used to calculate the final mean and standard error of mean (SEM). Data from flow cytometric analysis of cell phenotypes, results of which are shown in graphs, represent the mean and standard deviation (SD). Statistically significant differences between the infected and treated groups were calculated using one-way ANOVA followed by Tukey’s post hoc test. In the case of group analysis, two-way ANOVA was applied, followed by Bonferroni’s or Sidak’s multiple comparison analyses. Data were evaluated with GraphPad Prism (version 10.4.2.) (GraphPad Software, Inc., San Diego, CA, USA) and statistical differences in Figures are denoted as * *p* < 0.05, ** *p* < 0.01 and *** *p* < 0.001.

## 5. Conclusions

In conclusion, this study demonstrates that co-administration of ABZ+HLE significantly improves larvicidal efficacy in a murine model of *Mesocestoides vogae* infection. The combination therapy not only boosted the larval-killing effect of ABZ but also rebalanced the immune environment by enhancing Th1 responses and suppressing regulatory T and B cell subsets. Therefore, HLE represents a promising immunomodulatory supplement to chemotherapy in helminth infections. Additional studies are needed in order to explore the broader applicability of HLE as an adjuvant treatment in other helminthiases revealing the biological mechanism of action, to improve therapeutic strategies for parasite control and host protection.

## Data Availability

All data generated or analysed during this study are included in this article.

## Supplementary Materials

The following supporting information can be downloaded at: https://www.mdpi.com/article/10.3390/ijms26146994/s1.
