# Supplementary material for: Human Dialyzable Leukocyte Extract Enhances Albendazole Efficacy and Promotes Th1/Th2-Biased Lymphocyte and Antibody Responses in Peritoneal Cavity of Murine Model of Mesocestoides vogae Infection
Source: Int J Mol Sci. 2025 Jul 21;26(14):6994. doi: 10.3390/ijms26146994 (PMC12295987; doi:10.3390/ijms26146994)
Supplement: Supplementary file 1 [file ijms-26-06994-s001.zip › ijms-3733541-supplementary.pdf]

Table S1. List of oligonucleotides and their sequences.

Gene	Orientation	Sequence	Annealing temperature °C	Annealing time (sec)
GAPDH	forward	5′-AGGTCGGTGTGAACGGATTTG-3′	55.5	30
	reverse	5′-TGTAGACCATGTAGTTGAGGTCA-3′		
Tbet	forward	5′-GCCAGGGAACCGCTTATATG-3′	55.2	30
	reverse	5′-TGGAGAGACTGCAGGACGAT-3′		
GATA3	forward	5′-GAAGGCATCCAGACCCGAAAC-3′	55.2	40
	reverse	5′-ACCCATGGCGGTGACCATGC-3′		
Foxp3	forward	5′-AATAGTTCCTTCCCAGAG-3′	56.2	40
	reverse	5′-GATTTCATTGAGTGTCCT-3′		
CD9	forward	5′- CTC ATGATGCTGGTTGGTTTCC-3′	57.0	40
	reverse	5′- CTCTAGACCATTTCTCGGCTC C-3′		
Atf3	forward	5- AGCCTGGAGCAAAATGATGCT T-3′	53.5	45
	reverse	5- AGGTTAGCAAAATCCTCAAACAC-3′		
14-3-3	forward	5′- AAGAAGCAGGCTGGAACTCC-3′,	55.5	30
	reverse	5′- ACACCTCATTGCAGACCTCC-3′		
α-smooth actin	forward	5′-GTCCTACGAACTTCCCGACG-3′,	55.5	30
	reverse	5′-CGGCAGATTCCATACCCAGG-3′		
